# Pyrosequencing data analysis software: a useful tool for EGFR, KRAS, and BRAF mutation analysis

**DOI:** 10.1186/1746-1596-7-56

**Published:** 2012-05-28

**Authors:** Shanxiang Shen, Dahui Qin

**Affiliations:** 1 Department of Pathology, Moffitt Cancer Center, 12902 USF Magnolia Drive, Tampa, Florida, 33612, USA

**Keywords:** EGFR, KRAS, BRAF, Pyrosequencing, Software

## Abstract

**Background:**

Pyrosequencing is a new technology and can be used for mutation tests. However, its data analysis is a manual process and involves sophisticated algorithms. During this process, human errors may occur. A better way of analyzing pyrosequencing data is needed in clinical diagnostic laboratory. Computer software is potentially useful for pyrosequencing data analysis. We have developed such software, which is able to perform pyrosequencing mutation data analysis for epidermal growth factor receptor, Kirsten rat sarcoma viral oncogene homolog and v-raf murine sarcoma viral oncogene homolog B1. The input data for analysis includes the targeted nucleotide sequence, common mutations in the targeted sequence, pyrosequencing dispensing order, pyrogram peak order and peak heights. The output includes mutation type and percentage of mutant gene in the specimen.

**Results:**

The data from 1375 pyrosequencing test results were analyzed using the software in parallel with manual analysis. The software was able to generate correct results for all 1375 cases.

**Conclusion:**

The software developed is a useful molecular diagnostic tool for pyrosequencing mutation data analysis. This software can increase laboratory data analysis efficiency and reduce data analysis error rate.

**Virtual slides:**

The virtual slide(s) for this article can be found here: http://www.diagnosticpathology.diagnomx.eu/vs/1348911657684292.

## Background

Epidermal growth factor receptor (EGFR), Kirsten rat sarcoma viral oncogene homolog (KRAS) and v-raf murine sarcoma viral oncogene homolog B1(BRAF) are oncogenes, which may harbor mutations. Molecular diagnosis of these mutations is critical in making therapeutic decisions [1-8]. Pyrosequencing is a direct sequencing technology and can be used for detection of these mutations [9-14]. In our clinical molecular diagnostic laboratory, pyrosequencing is used for EGFR (codon 719, 746–753, 768, 790 and 858), KRAS (codon 12, 13 and 61) and BRAF (codon 600) mutation tests.

When compared to Sanger sequencing, pyrosequencing has several advantages. First of all, it has higher sensitivity. Sanger sequencing needs greater than 20 % of tumor load in a specimen to render a reliable result, while pyrosequencing can render a reliable result with 5 % tumor load. Therefore, pyrosequencing has higher sensitivity. Second, pyrosequencing is faster than Sanger sequencing. Third, pyrosequencing is more cost effective. One of the disadvantages of pyrosequencing is that it can only sequence a short length of nucleotide sequence. The other disadvantage is that pyrosequencing data analysis sometimes can be complex and challenging. The pyrosequencing data analysis for EGFR, KRAS and BRAF is a manual process. Pyrosequencing data output is a pyrogram. The pyrogram consists of a series of peaks with different peak heights, which reflect nucleotide sequence in a targeted DNA segment. Several variables need to be considered during pyrogram data analysis. These variables include the dispensing order, the pyrogram peak sequence, the peak heights, the wildtype sequence of the targeted gene, the possible mutations in a targeted gene, and the ratio of wildtype and mutant genes in a given specimen. Although pyrosequencing data analysis is relatively straight forward for some mutations, it can be complex for other mutations. Moreover, the ratio of wildtype and mutant gene copies varies case by case, which further complicates the pyrogram data analysis. Therefore pyrosequencing data analysis is a relatively sophisticated manual process, during which human errors can occur. We developed a computer software program that can facilitate the pyrogram data analysis.

## Methods

### Data

The pyrosequencing data from 1375 de-identified routine clinical mutation tests were analyzed, which is the total number of pyrosequencing tests performed in our lab from February, 2011 to December, 2011. The specimen DNA was extracted from unstained paraffin sections using QiaCube (Qiagen, Valencia, CA 91355) after manual micro-dissection.

### Pyrosequencing

The pyrosequencing for EGFR, KRAS and BRAF mutation was performed according to manufacture instructions (Qiagen, Valencia, CA 91355, [15-17]) with some modifications. Briefly, the DNA sequences of EGFR, KRAS and BRAF that may contain common mutations are selected as targeted sequences. The targeted sequences are amplified using PCR. Each PCR product is used as a template and is sequenced using pyrosequencing. The sequencing primer is designed to complement and hybridize to the sequence near the targeted mutation and it usually is within a few nucleotides. (Table 1 for the targeted sequences in EGFR, KRAS and BRAF). A unique dispensing order is designed for each targeted sequence, which is determined based on the targeted sequence and possible mutation(s) in the targeted sequence. (Table 2 shows the dispensing order for the targeted sequences in EGFR, KRAS and BRAF).

**Table 1:** Targeted Mutations and Sequences for EGFR, KRAS and BRAF mutations

**Targeted Mutations**	**Targeted Sequences**
EGFR exon 18, codon 719	DSCTCCGGTGC
EGFR exon 19 deletions	TATCAA[GGAATTAAGAGAAGC]AACATCTCCGAAAGCCA
EGFR exon 20, Codon 768	CAGCGTGGACAACCCCCACG
EGFR exon 20, Codon 790	ATCAYG
EGFR exon 21	CKGGCCAAACDGCTGGGT
KRAS codon 12 & 13	NNTGRCGTAGGC
KRAS codon 61 (reverse sequencing)	CTCDTGACCTG
BRAF codon 600 (reverse sequencing)	CWCTGTAG

Note: “D” could be A, T, U, or G (but not C); “S” could be C or G; “Y” could be C, T or U; “N” could any base; “R” could be A or G; “W”: T, U, or A. “[GGAATTAAGAGAAGC]” indicates deletion of the sequence.

**Table 2:** Targeted Mutations and Pyrosequencing dispensing order for EGFR, KRAS and BRAF mutations

**Targeted Mutations**	**Pyrosequencing Dispensing Orders**
EGFR exon 18, codon 719:	CATGTCACTCGTG
EGFR exon 19 deletions	CTATCACTGTCAGCTCGATCGTCATCGTCACGC
EGFR exon 20, Codon 768 and insertions	GCAGTACGTGTCGTGTACGTGACCACACTG
EGFR exon 20, Codon 790	GATTCATCTG
EGFR exon 21	ACGTGTCACATGTC
KRAS codon 12 & 13	ACTGTACGTGATCGTAGCAAGAG
KRAS codon 61 (reverse sequencing)	GCTCGATACGACCT
BRAF codon 600 (reverse sequencing)	TCGTATCTGTAG

During pyrosequencing, a sequence primer hybridizes to the targeted DNA template. Polymerase uses deoxyribonucleotide triphosphates (dNTPs) to synthesize a new DNA strand starting from 3’ end of sequence primer along the DNA template. The dNTPs are dispensed into the reaction tube one by one according to the dispensing order. When a dispensed dNTP is complementary to the nucleotide in the DNA template, the dNTP is incorporated into the newly synthesized DNA strand. At the same time, a pyrophosphate (PPi) is released. The released PPi is converted into adenosine triphosphate (ATP) by sulfurylase. The ATP is then used by luciferase to convert luciferin to oxyluciferin, during which visible light is generated in amounts that are proportional to the amount of ATP. The visible light is then captured and depicted as a peak in the pyrogram. In a pyrogram, peaks are labeled as A or C or G or T based on which dNTP is dispensed at the time. The peak height is proportional to the number of complementary base(s) in the template at the point of the dispensing.

**Figure 1:**
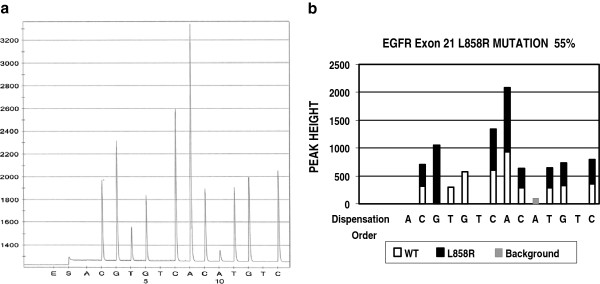
**The pyrogram of EGFR exon21 mutation and software analysis result.** Panel A is a pyrogram of EGFR L858R mutation. The small peak at the dispensation position 8 (A peak) is seen in all cases with similar peak height regardless of different tumor loads, therefore it is considered as non-specific noise. Panel B is the software data analysis of Panel A, indicating which peak or which portion of a peak is from either wildtype or mutant gene.

Figure 1A is an example of an EGFR exon 21 pyrosequencing result. The targeted sequence is CTGGCCAAA**CTG**CTGGGT. The bold CTG is codon 858 and is wildtype. During pyrosequencing, the instrument dispenses dNTPs one at a time in the dispensing order of ACGTGTCACATGTC. The first dispensed dNTP is deoxyadenosine alfa-thio triphosphate (dATPaS), which serves as a substitute of deoxyadenosine triphosphate (dATP). dATPaS is used as a substitute for the natural dATP because it can be used by the DNA polymerase to synthesize DNA, but is not recognized by the luciferase. Since there is no complement nucleotide for adenosine (A) at this position in the template, dATPaS is not incorporated and no visible light is generated. Therefore, there is no peak at this position (dispensing position 1) in the pyrogram (see position 1 in Figure 1A, which is labeled as ‘A’). The second dispensed dNTP is deoxycystidine triphosphate (dCTP). Since there is a complement nucleotide in the template for cystidine (C) at this position, dCTP is incorporated into newly synthesized DNA strand and a C peak is generated (see position 2 in Figure 1A, which is labeled as ‘C’). The third dispensed dNTP is deoxyguanosine triphosphate (dGTP) and a G peak is observed (see position 3 in Figure 1A, which is labeled as ‘G’), indicating that there is a complement nucleotide in the template. Since the wildtype EGFR exon 21 is supposed to have a thymidine (T) at this position, the G peak observed at this position indicates that there is a T to G mutation, which, in fact, is corresponding to the EGFR L858R mutation.

### Software development

The software was developed using Microsoft Excel and is designed to identify common mutations in EGFR, KRAS and BRAF respectively. The source of common mutations is http://www.sanger.ac.uk/genetics/CGP/cosmic/ (up to 6-6-2011). A portion of the test information is built into the software, which includes the targeted nucleotide sequence, common mutations and pyrosequencing dispensing order. Other test information needs to be input after testing, when test result raw data are available. The raw data include pyrogram peak sequences and peak heights. These data are copied and pasted into computer for software data analysis. The software analysis algorithm involves multiple steps, which can be illustrated as following, using EGFR mutation as an example. Step 1 is pattern recognition. In this step, the software compares the pyrogram peak with the known wildtype peak pattern that has been built into the software. This includes the comparison of peak sequence and peak heights of the test result to that of wildtype. If the resulted peak fits a wildtype peak pattern, the software will call it wildtype. If the resulted peak does not fit wildtype pattern, the software will compare it to the common mutant peak patterns that have been built into the software. If it fits one of the mutant peak patterns, the software will consider this mutant pattern as a candidate mutation. In the example shown in Figure 1, the peak pattern fits the EGFR L858R mutation. Therefore, the software will consider the L858R mutation as the candidate mutation and will do next step analysis. In the next step, the software will calculate the percentage of the candidate mutant gene in the specimen, using a built-in formula. In case of EGFR exon 21 L858R mutation, the formula is as following:

[1/3 x A/B + (1-C/B) + (1-D/E)]/3x100.

“A” is the peak height of the second peak (which is labeled as ‘G’ at the dispensing position 3 in Figure 1).

“B” is the average peak height of the reference peaks, each of which is resulted from a single nucleotide incorporation. The reference peaks include the first peak (which is labeled as ‘C’ at the dispensing position 2 in Figure 1), the seventh peak (which is labeled as ‘C’ at the dispensing position 9), the eighth peak (which labeled as ‘T’ at the dispensing position 11), the ninth peak (which is labeled as ‘G’ at the dispensing position 12) and the tenth peak (which is labeled as ‘C’ at the dispensing position 14).

“C” is the peak height of the third peak (which is labeled as ‘T’ at the dispensing position 4 in Figure 1).

“D” is the peak height of the fourth peak (which is labeled as ‘G’ at the dispensing position 5 in Figure 1).

“E” is the peak height of the fifth peak (which is labeled as ‘C’ at the dispensing position 7 in Figure 1).

A particular formula for each mutation is programmed into the software since each mutation has its unique pyrogram peak pattern. If the calculated percentage of mutant component is higher than 5 %, the software will call it mutant. If the percentage is lower than 5 %, the software will not call it mutant since our validated test sensitivity is set to 5 %. The software analysis result is shown in Figure 1B. It indicates that the second peak G is from a mutant; the third and fourth peaks T and G are from wildtype; the rest of the peaks represent a mixture of both mutant and wildtype and the 55 % of the targeted nucleotide sequence in this specimen is from the mutant gene.

**Figure 2:**
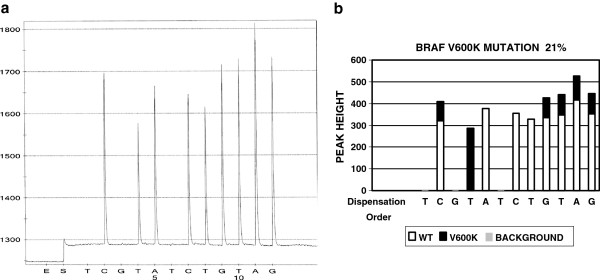
**The pyrogram of BRAF V600K mutation and software analysis result.** Panel A is a pyrogram of BRAF V600K mutation. Panel B is the software analysis of Panel A, indicating which peak or which portion of a peak is from either wildtype or mutant gene.

The software developed as such is a working draft. The working draft needs to be fine-tuned to accommodate normal variations in lab tests. The fine-tuning is the process of testing the software draft on real cases. Since test results have normal variations, the working draft software usually recognizes some mutations and misses others. Therefore, the parameters in the software draft need to be adjusted based on real case test data. For example, we had a BRAF mutation case in the early stage of our software development. The BRAF test pyrogram is shown in Figure 2A. When the working draft software was used to analyze this pyrogram, the result was positive for BRAF V600E mutation (data not shown). However, this specimen is actually positive for V600K mutation. The working draft misinterpreted the result because it failed to distinguish the subtle changes of the peak heights in the fourth and fifth peaks (C and T) from normal variations. In this case, the peak C and T are slightly lower than normal C and T. When this occurs together with a second peak T, it indicates a BRAF V600K mutation. The working draft is modified accordingly, adding peak height of C peak as a variable. After such fine-tuning, the software is able to recognize this case as V600K mutation (Figure 2B). A total of 490 test results (121 for EGFR, 149 for KRAS, and 220 for BRAF) were used during the fine-tuning process.

**Figure 3:**
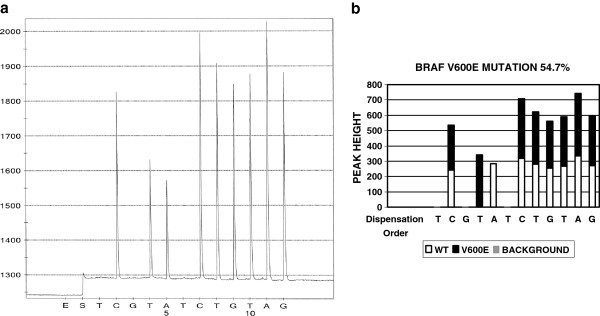
**The pyrogram of BRAF V600E mutation and software analysis result.** Panel A is a pyrogram of BRAF V600E mutation. Panel B is the software analysis of Panel A, indicating which peak or which portion of a peak is from either wildtype or mutant gene.

Non-specific peaks (background noise and artifact) are also present in pyrosequencing. To determine if a small peak is artifact/non-specific peak, a cut off threshold is needed. In this project, two times unexpected peak height average was used as the cut off threshold. The unexpected peaks are defined as the peaks observed at dispensing positions where no peak is expected to be present in either wild type or in common mutants. Using BRAF mutation as an example (see pyrograms in Figures 2A and 3A), the dispensing order is TCGTATCTGTAG and usually no peak is expected at the dispensing positions 1, 3, and 6. Any small peaks at these positions are considered as unexpected peaks. The heights of these unexpected peaks are used to calculate the unexpected peak height average. Two times this unexpected peak height average was used as the cut off. Any peaks with the peak height lower than the cut off are considered as non-specific peaks (artifact).

### EGFR, BRAF and KRAS Mutation Test Analysis

EGFR, BRAF and KRAS mutation tests are routine clinical tests in our clinical molecular lab using pyrosequencing. The raw data is manually analyzed by two lab staff members independently before a result is issued. For this project, the software was used to independently analyze the pyrosequencing data in parallel with manual analysis. The manual analysis results were compared to the software generated results.

## Results

1. EGFR Mutation Data Analysis:

EGFR codon 719, 746–753, 768, 790 and 858 were analyzed for this project. Figure 1A is a pyrogram of exon 21 L858R mutation. The software analysis indicates that there is a L858R mutation. 55 % of gene sequence in this specimen is from mutated gene (Figure 1B). 355 test results have been analyzed by manual and software analysis. The results are listed in Table 3. The software identified 78 positive cases and the manual review identified 76 positive cases. In two occasions, exon 19 deletions were overlooked by one reviewer in manual analysis, but picked up by the software.

2. BRAF Mutation Data Analysis:

**Table 3:** Comparison of Manual and Software Data Analysis Results for EGFR mutations

	**Positive**	**Negative**
Manual analysis	76	279
Software analysis	78	277

Positive: indicating that the specimen is positive for either exon 18, 19, 20 or 21. Negative: indicating that no EGFR mutation is identified in the targeted sequences.

Most common BRAF mutations occur at codon 600. Therefore, it is the targeted sequence in this assay. Different mutation variants have been reported at this codon, e.g. V600E, V600K and V600R. The sequence to be analyzed is ACA**GTG** in wild type. The bold GTG is codon 600. Since the sequencing primer is a reverse sequencing primer, the actual sequence that is analyzed is the reverse and complement of ACAGTG, hence CACTGT. The resulted pyrogram is seen in Figure 3A indicating a V600E mutation. The software analysis result in Figure 3B reaches the same conclusion. 613 test results were analyzed by both manual and software analysis and the results are listed in Table 4. One V600K mutation was overlooked in the first round of manual analysis and picked up by software analysis.

3. KRAS Mutation Data Analysis:

**Table 4:** Comparison of Manual and Software Data Analysis Results for BRAF mutations

	**Positive**	**Negative**
Manual analysis	202	411
Software analysis	203	410

Positive: indicating that the specimen is positive for either V600E, V600K or other codon 600 mutations. Negative: indicating that no BRAF codon 600 mutation is identified.

**Figure 4:**
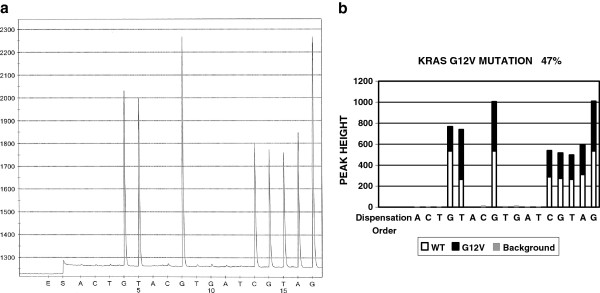
**The pyrogram of KRAS G12V mutation and software analysis result.** Panel A is a pyrogram of KRAS G12V mutation. Panel B is the software analysis of Panel A, indicating which peak or which portion of a peak is from either wildtype or mutant gene.

KRAS mutations may involve codon 12, 13, and 61. Each codon may have more than one mutation variant. Figure 4A shows a pyrogram of the G12C mutation and Figure 4B is the software analysis result of the same case, indicating the G12C mutation with 47 % targeted sequence containing mutated gene in this specimen. 407 test results were analyzed by both manual and software analysis and the results are listed in Table 5. One mutation (G13D) was overlooked by the first round of manual analysis and picked up by software analysis.

**Table 5:** Comparison of Manual and Software Data Analysis Results for KRAS mutations

	**Positive**	**Negative**
Manual analysis	167	240
Software analysis	168	239

Positive: indicating that the specimen is positive for either codon 12, 13, or 61. Negative: indicating that no KRAS mutation is identified in the targeted sequences.

Among a total of 1375 tests analyzed, one-reviewer’s manual analysis identified 347 positive results and 1028 negative results. The software identified 351 positive results and 1024 negative results, which was confirmed by a second reviewer. When the manual analysis result is compared with software analysis result, Chi square equals 0.061 and the two-tailed p value equals 0.8046. The software may serve as a useful tool for quality control purpose while the difference between the two detection rates are of no statistic significance.

## Discussion

**Figure 5:**
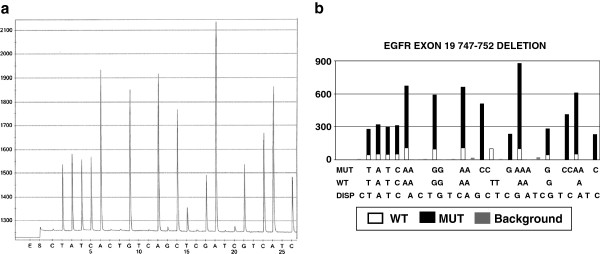
**The pyrogram of EGFR exon19 747–752 deletion and software analysis result.** Panel A is a pyrogram of EGFR exon 19 deletion between codon 747 and 752. Panel B is the software data analysis of Panel A, indicating which peak or which portion of a peak is from either wildtype or mutant nucleotide sequence. ‘Mut’ stands for mutant sequence nucleotides that contribute to the corresponding peak. ‘WT” stands for wildtype sequence nucleotides that contribute to the corresponding peak. ‘DISP” stands for dispensing order during pyrosequencing.

The manual analysis of pyrosequencing data sometimes is a complex process. Human error can occur during data analysis. The main errors in manual analysis occur in three aspects. The first is due to the complexity of some mutations. Among the tests that are performed in our lab, EGFR exon 19 mutations are the most complex. The mutations in EGFR exon 19 are usually deletions and there are many different deletions. Ten of these deletions are more common than others. Each deletion will generate a unique pyrogram pattern with different peaks reflecting nucleotide sequence. Both wild type and mutant nucleotide sequence may contribute to a certain peak. Therefore, each pyrogram peak may reflect wild type nucleotide sequence or mutant one, or both. The nucleotides contributing to a certain peak may come from different codons of wild type and mutant sequences due to deletion. Each pyrogram pattern may further vary due to different tumor load in each individual case. All of these variations make manual analysis difficult. However, such complexity doesn’t pose a problem for computer software analysis. Once all of these possible different combinations have been programmed into the software, the computer can sort through these possible different combinations in a rapid fashion. Figure 5A is the pyrogram of EGFR exon 19 deletion between codon 747 and 752. Figure 5B is the software analysis result. It shows that both wildtype and mutant gene nucleotide(s) contribute to different pyrogram peaks. The second type of error in manual analysis is overlooking subtle mutation changes. An example is BRAF V600K mutation. The targeted sequence of BRAF in reverse sequencing is CACTGTAG. The dispensing order is TCGTATCTGTAG (Figure 2A and 2B). In the case of the V600K mutation, apart from mutant peak T (second peak at dispensing position 4), the fourth peak C and fifth peak T (at the dispensing position 7 and 8) are lower than normal. One V600K was missed because the second peak T distracted the data reviewer. Consequently, the subtle changes in the fourth peak C and fifth peak T were overlooked. The third type of error in manual analysis is overlooking less common mutant peaks. For example, in KRAS data analysis, codon 12 mutation is more common. The data reviewer may focus on codon 12 changes and overlook the changes in codon 13. In this project, a KRAS G13D was missed for this reason.

The main error in computerized analysis is that suboptimal parameters are used to build the software for certain mutations. For example, in the case of the V600K mutation, the parameter for the lower fourth peak C was initially set up as “the height of fourth peak C is lower than 95 % of the average peak height of equivalent normal peaks. In this case, the dispensing order is TCGTATCTGTAG. The sixth, seventh and ninth peaks (which are labeled as G, T and G at the dispensing position of 9, 10 and 12) are used to calculate average normal peak height. During the testing process, it was realized that although such settings can recognize some V600K mutations, but will occasionally misinterpret some V600K cases as V600E. Therefore, the parameter was modified so that instead of using only the fourth peak C, both the fourth peak C and fifth peak T are used in the calculation. Moreover, instead of using “95 % of the average”, “less than two standard deviations of the average” is used. The modified software was tested and was able to interpret the data correctly. It appears that standard deviation reflects normal variation better than an arbitrary 95 %. Such modification is part of fine-tuning process of this software development.

Normally, two individuals will check sequencing results to minimize the human error. In this project, we used our software to check a total of 1375 test results (355 EGFR, 613 BRAF and 407 KRAS). The software was able to pick up 4 errors from the first round of manual analysis, which were also picked up by the second reviewer. The results indicate that the pyrosequencing data analysis software can be used as another layer of quality control.

The pattern recognition concept has been used to generate software for pyrosequencing data analysis. For example, Joakim Lundeber et al have used it for SNPs in chromosome 9 [18]. Pyrosequencing software from Qiagen can provide pyrogram patterns for pure homozygous and heterozygous results of most common mutations in EGFR, KRAS and BRAF [15-17]. A recent software, Pyromaker can provide simulated pyrogram patterns with different percentages of tumor cells [19]. The software developed in our lab is able to analyze real case data. Real case data can be input into our software and the output result will indicate what mutation type and percentage of mutant gene in the specimen. Our software also provides more extensive coverage for various mutations in EGFR, KRAS and BRAF. For example, it has been tailored in such a way so that it can distinguish BRAF V600E, V600K and V600R mutations. It can also distinguish different common variants of EGFR exon 19 deletions. Our software is also fine-tuned to accommodate normal variations in clinical mutation tests. Such features of the software make it a practical tool for pyrosequencing data analysis of real cases.

Based on our literature search using keywords, such as pyrosequencing, software, EGFR, KRAS and BRAF, our software is a unique program developed for EGFR, KRAS and BRAF pyrosequencing data analysis.

The software is designed and fine-tuned by our lab staff members and the software can only be as good as our lab staff members. However, the lab staff’s knowledge and experiences can be built into the software during the fine-tuning process. With such collective wisdom, the software may perform better than one staff member performing manual analysis. Moreover, the software can work more consistently and objectively than a human does, which makes it a valuable quality control tool.

The fine-tuning is also an ongoing training process for the software, especially for rare mutations. Our first stage fine-tuning used the data from 490 mutation test results. This process will continue in our lab as we analyze more cases. The molecular lab staff serves as trainers. Whenever a new mutation is misread by the software, our lab will update the software to cover the new mutation. We will adjust analysis parameters so that the software will be able to recognize the new mutations correctly without losing specificity. Our software is an open system. More coverage of mutations can be added to the software when needed.

## Conclusion

The pyrosequencing data analysis software developed is a useful tool. It will tremendously increase the efficiency and consistency of pyrosequencing data analysis.

## Abbreviations

EGFR, Epidermal growth factor receptor; KRAS, Kirsten rat sarcoma viral oncogene homolog; BRAF, v-raf murine sarcoma viral oncogene homolog B1 mutations.

## Competing interest

No competing interest is identified for the authors.

## Authors’ contributions

DQ performed manual pyrosequencing data analysis, participated in designing the software algorithm flow chart and drafted the manuscript. SS carried out the pyrosequencing test, developed the software and helped in manuscript preparation. All authors read and approved the final manuscript.
